# Lack of research on efficacy of virtual reality exposure therapy (VRET) for anxiety disorders in children and adolescents: A systematic review

**DOI:** 10.1007/s40211-020-00349-7

**Published:** 2020-05-05

**Authors:** Oswald D. Kothgassner, Anna Felnhofer

**Affiliations:** 1 Department of Child and Adolescent Psychiatry, Medical University of Vienna, Waehringer Guertel 18–20, 1090 Vienna, Austria; 2 Department of Pediatrics and Adolescent Medicine, Medical University of Vienna, Waehringer Guertel 18–20, 1090 Vienna, Austria

**Keywords:** Virtual reality, Anxiety disorders, Specific phobia, Exposure treatment, Youth, Virtuelle Realität, Angststörungen, Spezifische Phobie, Expositionsbehandlung, Jugend

## Abstract

Anxiety disorders are one of the most prevalent mental disorders in children and adolescents which may effectively be treated by several forms of exposure therapy. An emerging approach to exposure is virtual reality exposure therapy (VRET), but a literature search synthesis focusing specifically on the use of VRET in children and adolescents is still lacking. This systematic review sets out to provide an overview concerning VRET for the treatment of anxiety disorders in this age group. Four published trials covering an overall sample of 100 participants between the ages of 8 and 16 years were found during a systematic literature search and were included in the current review. Results reveal that participants show clinical improvements regarding anxiety symptoms after VRET. Nevertheless, the high potential of virtual reality as a tool for treating children and adolescents with anxiety disorders is contrasted by a considerable lack of controlled trials. Despite the evidence of VRET in adult samples, there is a need for more research with younger cohorts in order to be able to support this promising field of application.

## Abstract (de)

Angststörungen gehören zu den häufigsten psychischen Störungen im Kindes- und Jugendalter, die durch verschiedene Formen der Expositionstherapie wirksam behandelt werden können. Ein neuer Ansatz zur Exposition ist die VRET (Virtual Reality Exposure Therapy), welche zunehmend in der Behandlung von Angststörungen gute Erfolge erzielt. Eine Aufarbeitung der Literatur dazu aus dem Kinder- und Jugendbereich ist allerdings noch ausstehend. Dieser systematische Review soll daher einen Überblick über die VRET als Behandlungsmethode für Angststörungen in dieser Altersgruppe geben. Vier veröffentlichte Studien, die insgesamt eine Stichprobe von 100 Teilnehmenden im Alter von 8–16 Jahren umfassen, wurden mithilfe einer systematischen Literaturrecherche identifiziert und in die hier vorgestellte Übersicht aufgenommen. Die Ergebnisse belegen, dass sich bei den Teilnehmenden nach VRET klinische Verbesserungen der Angstsymptome zeigen. Dem hohen Potenzial der virtuellen Realität als einem Instrument zur Behandlung von Kindern und Jugendlichen mit Angststörungen steht allerdings ein erheblicher Mangel an kontrollierten Studien gegenüber. Trotz der Evidenz für VRET in Erwachsenenstichproben besteht weiterer Forschungsbedarf mit jüngeren Kohorten, um dieses vielversprechende Anwendungsfeld unterstützen zu können.

## Introduction

Anxiety disorders are considered to be one of the most prevalent clusters of disorders in children and adolescents with a lifetime prevalence of 28.8% [12]. Recently, a school-based study in Austria [26, 28] revealed that the lifetime prevalence of anxiety disorders was up to 15.5%, with an estimated prevalence of 7.3% for specific phobias (SPH), 3.5% for social anxiety disorders (SAD), and 1% for panic disorders (PD). Moreover, the prevalence was even higher in a population from mental health services, ranging between 9.5 and 16.0% for SPH, SAD and PD [26]. Another recent study on a mental hospital cohort in Austria reported that particularly female adolescents suffer more often from anxiety disorders [22].

Exposure therapy has been supported as a first-line evidence-based treatment for most anxiety disorders, like SPH, SAD, PD, as well as for posttraumatic stress disorders (PTSD), and obsessive–compulsive disorders (OCD) [3]. Accordingly, research indicates that exposure therapy can also be considered as a highly effective and efficacious treatment for children and adolescents with anxiety disorders [27]. This therapy approach sets out to—on the one hand—activate the phobic structure upon exposure to the feared stimulus, and to—on the other hand—achieve symptom reduction by habituation following repeated confrontation with the according stimulus (*in vivo* or *in sensu*) [8]. These two classical methods of exposure therapy, *in vivo* (exposure to a real stimulus) and *in sensu* (exposure to an imaginal stimulus), were amended by a technology-mediated form of exposure: virtual reality exposure therapy (VRET). In this approach, phobias are treated using a head-mounted device to present a computer-based feared virtual environment or a feared virtual stimulus. Recent studies [13, 29] show that exposure to a feared stimulus in virtual reality (VR) provokes levels of anxiety as well as physiological responses which are comparable to those induced by an exposure *in vivo*. Furthermore, experiences in VR influence emotional states and physiological responses in subsequent real-life interactions (e.g., subsequent emotional and physiological reactivity to real-life stressors; prolonged prosocial behavior) [6, 14, 15]. Additionally, there is a significant difference in the physiological activation between patients and healthy controls during exposure to a feared stimulus in VR (e.g., Felnhofer et al. [7]).

In sum, VRET is a valuable and effective treatment tool for anxiety disorders. It is also suggested by S3 guidelines as an evidence-based method particularly for specific phobias [4]. Accordingly, a recent meta-analysis by Carl et al. [5] including 30 randomized controlled trials (RCTs) supports the efficacy of VRET. The authors found that half of these studies compared VRET with cognitive behavioral therapy (CBT) -based in vivo exposure (IVE) revealing no differences in effect sizes between VRET and IVE. While these effects are especially true for SPH, SAD, and PD, another recent meta-analysis [16] showed that they could not be replicated for VRET in PTSD patients. In this paper, only 5 studies that compared VRET with an active comparator were identified. However, none of these active groups encompassed in vivo exposure or other first-line treatments as suggested by several guidelines (e.g., American Psychological Association [1]); hence, there is no clear evidence that VRET may also be effectively applied in PTSD patients. Apart from these limitations, the most recent meta-analyses on anxiety disorders [5, 16] did not include any trials focusing on VRET for SPH, SAD, PD, or PTSD in children or adolescents. All studies applied VRET only in adult patients. Hence, despite the large potential and efficacy of using VRET for the treatment of anxiety disorders (e.g., Carl et al. [5]), there is still a lack of systematic analyses of the current literature regarding the use of VRET in children and adolescents with anxiety disorders. Thus, this systematic review set out to provide a comprehensive overview of existing research on VRET for children and adolescents suffering from anxiety disorders including PTSD.

## Methods

**Fig. 1:**
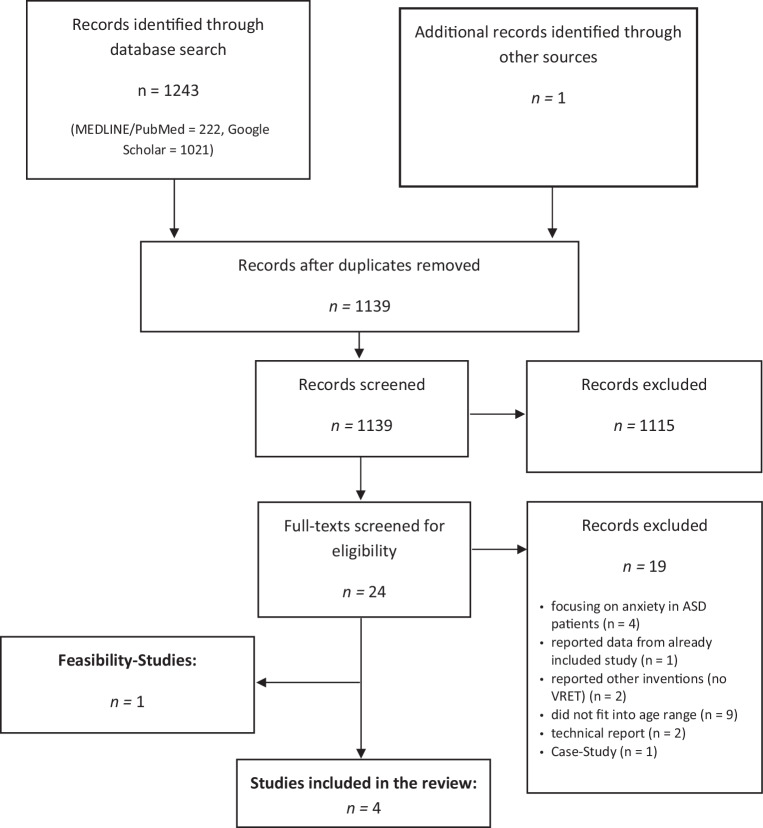
PRISMA flowchart of screening, exclusion and inclusion criteria. *VRET* virtual reality exposure therapy, *ASD* autism spectrum disorder

We selected trials from MEDLINE/PubMed and Google Scholar using the keywords ‘VRET OR Virtual Reality Exposure Therapy AND anxiety disorders’ in combination with terms ‘therapy’, ‘treatment’ and ‘children’ or ‘adolescents’ or ‘youth’. Alternatively, we were searching for keywords ‘VR OR Virtual Reality AND anxiety disorders’ in combination with the above mentioned terms, as well as for ‘VRET OR Virtual Reality Exposure Therapy AND phobia’; ‘VRET OR Virtual Reality Exposure Therapy AND trauma’. We set the time range from the beginning of database records until January 2020. Studies were included if they reported at least one group with VR treatment. All control group interventions were included in the systematic review. However, all included studies were imposed with the restriction that all participants had to be less than 19 years of age. Feasibility studies lacking an overall evaluation of the treatment were registered and reported, but not included in the main analysis. Additionally, Google Scholar alerts were enabled to ensure inclusion of articles in press. Exclusion of documents occurred at each stage (see Fig. 1 for PRISMA flow diagram). Final inclusion and exclusion decisions were based on the following criteria:

- *Participants*: Individuals with an age less than 19 years of age and anxiety disorder/subclinical anxiety. Studies focusing on anxiety symptoms in developmental disorders, or autism spectrum disorder (ASD) were excluded due to comparison issues with isolated anxiety disorders.
- *Intervention*: Virtual reality exposure therapy for anxiety disorders (SPH, SAD, PD, PTSD).
- *Comparison*: Studies with and without control groups were included. Therefore, pre–post evaluations or randomized controlled trials (RCTs) were included. Studies without an evaluation of the treatment effects *per se* were only reported as feasibility studies, but were not included in the main analysis.
- *Outcomes*: Studies reported at least a symptom severity score before and after the intervention.

The title, abstract, and manuscript of each study were examined by both authors independently. Risk of bias for each study based on the AHRQ Method Guide for Comparative Effectiveness Reviews [25] was assessed for each included study. Studies were rated according to categories regarding randomization, selection and attrition bias, confounding bias and measurement bias. Based on these predefined criteria, all studies were assessed with regard to low, moderate, or high risk of bias. We determined that inappropriate methods of randomization, not controlling for confounding factors such as high attrition ≥40% or differential loss ≥30% and problems in participant selection are reasons for high risk of bias ratings. Low risk of bias was predefined by appropriate randomization methods (e.g., computer-generated random allocation), low attrition <20% or differential loss <5%, no significant baseline differences among groups regarding primary outcome measures and inclusion/exclusion criteria, adequate control of confounders, use of validated assessments for outcome measures. Furthermore, we rated overall strength of evidence (SOE) according to Owens et al. [24] for all included studies as displayed in Table 1.

**Table 1:** Characteristics of the four studies included in the systematic review and one feasibility study

Study	Population	Control	Main outcome for review	*n* (VRET)	*n* (Control)	Age in years	Female %	Treatment duration	Eligibility	SOE	Main results
**RCTs**											
Gutiérrez-Maldonado et al. (2009) [10]	*School phobia*	RCT, WL	School-based fears (IME), General fears questionnaire (FSSC-R)	18	18	10 to 15	63.9	8 sessions	*Inclusion:* Cut-off criteria IME ≥30/50, FSSC‑R ≥150 *Exclusion:* other mental health problems, school refusal, antisocial behavior	Moderate	VRET was able to reduce the severity of reported school-based fears and showed better outcomes than WL (r = 0.64 for pre–post treatment comparison)
											VRET has no influence on general self-reported anxiety
St-Jacques et al. (2010) [23]	*Arachnophobia*	RCT, IVE	Spider Phobia (SPBQ, BAT)	17	14	8 to 15	83.9	5 sessions	*Inclusion:* Clinical interview with children and parent, criteria according to DSM-IV *Exclusion:* current psychological treatment/medication, other psychiatric disorder or severe medical condition	Moderate	VRET showed comparable reductions in the self-reported fear of spiders as IVE (η^2^ = 0.56 for pre–post treatment comparison)
											Differences between both conditions concerning BAT are reported at baseline (pre-measure)
											VRET did not increase motivation toward treatment
											Reported some adverse effects (children reported that they got “stuck in the headset”)
**Evaluations**											
Kahlon et al. (2019) [11]	*Public Speaking Anxiety*	Pre–Post evaluation, no control	Public Speaking Anxiety (PSAS)	27	N/A	13 to 16	78.0	90 min single session	*Inclusion:* 13–16 years of age, PSAS (observed range: 46–73, possible range 17–85) and functional impairment *Exclusion:* ongoing psychotherapy, use of benzodiazepines, lack of stereoscopic vision	Moderate	VRET showed significant decrease in self-reported Public Speaking Anxiety compared to pre-treatment (Cohens d = 1.53)
											Only small increase in heart rate during exposure
Servera et al. (2019) [21]	*Fear of Darkness*	Pre–Post evaluation, no control	Fear of Darkness (EMO)	6	N/A	8 to 12	50.0	6 to 8 sessions	*Inclusion: *8–12 years of age, EMO proxy rating ≥7	Low	Overall, VRET significantly reduced the fear of darkness reported by proxy compared to pretreatment baseline level (r = 0.50)
											VRET did not work in 1/3 of participants
**Feasibility-Study**											
Parrish et al. (2016) [19]	*Social Anxiety Disorder*	Feasibility Study, without evaluation, comparing SAD to Non-SAD		21	20	13 to 18	65.9	N/A	*Inclusion* 13–18 years of age, Cut-off criteria LSAS ≥29.5 *Exclusion: *other psychiatric diagnosis, alcohol/drug dependency, use of psychotropic medications, living in the same household than another participant, fear of closed spaces or inability to wear head-mounted display, specific health concerns, or pregnancy	N/A	VRET appears feasible for adolescents with SAD
											Adolescents with SAD showed differences to non-SAD regarding SUDs during public speaking

*VRET *virtual reality exposure therapy*, BAT* Behavioral Approach Test, *EMO* Escala de Evaluación del Miedo a la Oscuridad/Assessment Scale of Fear of Darkness, *FSSC‑R* Fear Survey Schedule for Children—Revised, *IME* School-Related Fears Inventory, *IVE* In vivo exposure treatment, *LSAS* Liebowitz Social Anxiety Scale for Children and Adolescents, *PSAS* Public Speaking Anxiety Scale, *SPBQ* Spider Phobia Beliefs Questionnaire, *SOE* Strength of Evidence, *SUDS* Subjective Units of Distress Scale, *WL* Waiting list, *RCT* randomized controlled trial

## Results

**Fig. 2:**
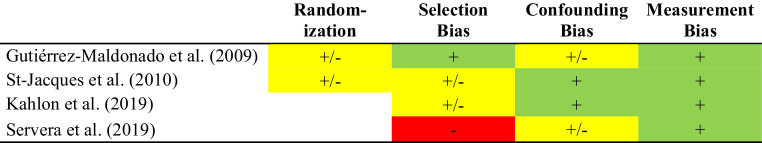
Risk of bias assessment [10, 11, 21, 23]. + low risk of bias (*green*), +/− moderate risk of bias (*yellow*), − high risk of bias (*red*)

The initial search following PRISMA Guidelines yielded 873 results. Titles and abstracts were screened for eligibility and full-text manuscripts were obtained (Fig. 1). After screening, k = 4 studies covering 100 participants (VRET-RCT: *n* = 35/control conditions: *n* = 32; pre–post evaluations: *n* = 33) were identified and included in the current review (see Table 1 for study characteristics). One feasibility study [19] was found without pre–post evaluation of the training. Our review suggests that both RCT studies [10, 23] showed moderate risk of bias. Pre–post evaluations showed low–moderate risk of bias. A detailed report about risk of bias in the specific domains of each study is presented in Fig. 2. Both RCTs reported an efficacy of VRET (Gutiérrez-Maldonado et al. [10] for School Phobia, St-Jacques et al. [23] for Arachnophobia). While Gutiérrez-Maldonado et al. [10] found a superior effect of VRET over a waitlist (WL) control group as assessed with self-report measures, the study by St-Jacques et al. [23] showed that the reported fear of spiders was reduced after VRET and IVE, but there was no differences between the two groups posttreatment. Both studies report large effect sizes concerning the effect of treatment. Kahlon et al. [11] and Servera et al. [21] both found large effect sizes for the significant differences between pretreatment and posttreatment measures regarding an improvement of self-reported public speaking anxiety symptoms as well as for fear of darkness levels reported by parents.

### Age and gender

All studies reported a relatively broad age range covering 8 to 16 years of age. Most studies had a definite trend toward female gender, except for Servera et al. [21] who reported a 1:1 proportion, yet had a small sample (*n* = 6).

### Adverse effects

Half of the included studies [11, 23] assessed the potential risk of simulator sickness. St-Jacques et al. [23] reported that child users were afraid of getting “stuck in the headset” and of seeing something “scary” like in a “horror movie”. Another trial [11] did not report on their assessment of adverse effects during VRET intervention.

### Sessions, attrition, and treatment response

The number of treatment sessions ranged between 5 and 8 sessions in most studies. There was only one study [11] with a single session covering 90 min. The duration of sessions in the other studies varied from 20–40 min [10, 21] up to 60 min [23]. Most studies reported a minimal loss of participants between pre- and posttreatment. However, the study by Servera et al. [21] had a higher attrition rate with a final sample of only 6 patients (initially, 37 patients were selected, 10 were randomized and started treatment), who were treated by nonexpert students. Moreover, the authors report that 2 patients in the final sample did not respond to the treatment at all.

### Feasibility studies

There was only one feasibility study [19] without an evaluation of the treatment, but instead assessing differences between adolescents with and without SAD regarding subjective units of distress (SUDs) when exposed to different virtual environments. This study concludes that VR environments simulating interpersonal interactions such as public speaking or visiting a party are able to provoke specific reactions of distress as well as acceptable levels of presence; the latter has previously been defined as an experience of nonmediation and a resulting sense of actually being there in the artificial environment (see Lombard and Ditton [17] for details). Accordingly, the participating adolescents with SAD stated a good acceptability of VR in their post-debriefing interview and described the simulations as “real” or “normal”. Hence, the authors concluded that good acceptability, high presence and higher levels of distress during the scenario demonstrated the suitability of VR for treating SAD patients.

## Discussion

Based on the lack of research syntheses regarding the use of VRET in children and adolescents with anxiety disorders, the current systematic review set out to provide an overview of the existing literature. The search yielded only two RCTs, two pre–post evaluation studies, and one feasibility study. Preliminary results support the notion that VRET may constitute an effective treatment not only in adults (see Carl et al. [5]), but also in children and adolescents: one study showed better treatment outcomes than WL controls [10], another found a comparable reduction of symptom severity in VRET and IVE [23]. Similarly, both evaluation studies [11, 21] reported considerable improvements in key symptoms posttreatment, and the feasibility study [19] concluded that VRET is feasible in adolescents with SAD.

Despite these encouraging results, research on VRET in children and adolescents with anxiety disorders is still scarce at best. More studies are needed, in particular RCTs, to support the preliminary finding that VRET may indeed be efficacious in younger populations. Also, the current evidence focuses mainly on specific phobias in children and adolescents (i.e., school phobia, arachnophobia, fear of darkness) and only marginally considers SAD. In comparison, no studies were found on the use of VRET in young patients with panic disorders or with PTSD. Particularly in light of the considerably high lifetime prevalence of PTSD among children and adolescents [18], VRET may provide a useful means of trauma treatment. As demonstrated by a recent meta-analysis [16], VRET already shows promising results compared to other treatments in adult populations.

Overall, generalization across different age groups and developmental stages are difficult or even impossible, particularly when considering a technology-based treatment method such as VR. For example, St-Jacques et al. [23] recorded adverse effects which had not previously been observed in adult samples using VR. Some of their participants reported they were afraid of the virtual environment and feared getting “stuck in the headset”, a finding which the authors discuss to be possibly related to the fact that children—due to their still developing ability to think abstractly—may not reflect in the same way on VR as adults who may more readily arrive at the estimation that virtual stimuli are less threatening than their in vivo counterpart. Furthermore, children’s motivation to use VR (see St-Jacques et al. [23]) may differ substantially from the motivation observed in adults, who have generally been found to be more willing to choose exposure to virtual stimuli over in vivo exposure [9]. Also, negative effects of VRET such as cybersickness may show age-related patterns. For instance, it has previously been observed that older adults suffer from more severe cybersickness symptoms than younger users of VR [2].

In sum, however, additional evidence is needed to learn more about specific needs and problems with VRET in children and adolescents. Especially, motivational factors should be reflected with great care and put in relation with developmental stages as well as according cognitive capacities (e.g., Piaget [20]). Therefore, age-specific effects regarding a potential selective efficacy—particularly in younger cohorts—need to be investigated in future studies (Servera et al. [21]).

Generally, the potential of VR in treating children and adolescents with anxiety disorders is contrasted by a considerable lack of RCTs. Results of the current review suggest a potential benefit of using VRET in younger cohorts and clearly call for more evidence to support this promising field of application.
